# Precision Targeted Mutagenesis via Cas9 Paired Nickases in Rice

**DOI:** 10.1093/pcp/pcw049

**Published:** 2016-03-02

**Authors:** Masafumi Mikami, Seiichi Toki, Masaki Endo

**Affiliations:** ^1^Graduate School of Nanobioscience, Yokohama City University, 22-2 Seto, Yokohama, Kanagawa, 236-0027 Japan; ^2^Plant Genome Engineering Research Unit, Agrogenomics Research Center, National Institute of Agrobiological Sciences, 2-1-2 Kannondai, Tsukuba, Ibaraki, 305-8602 Japan; ^3^Kihara Institute for Biological Research, Yokohama City University, 641-12 Maioka-cho, Yokohama, Kanagawa, 244-0813 Japan

**Keywords:** CRISPR/Cas9, Genome editing, Off-target, Rice, Targeted mutagenesis

## Abstract

Recent reports of CRISPR- (clustered regularly interspaced short palindromic repeats)/Cas9 (CRISPR-associated protein 9) mediated heritable mutagenesis in plants highlight the need for accuracy of the mutagenesis directed by this system. Off-target mutations are an important issue when considering functional gene analysis, as well as the molecular breeding of crop plants with large genome size, i.e. with many duplicated genes, and where the whole-genome sequence is still lacking. In mammals, off-target mutations can be suppressed by using Cas9 paired nickases together with paired guide RNAs (gRNAs). However, the performance of Cas9 paired nickases has not yet been fully assessed in plants. Here, we analyzed on- and off-target mutation frequency in rice calli and regenerated plants using Cas9 nuclease or Cas9 nickase with paired gRNAs. When Cas9 paired nickases were used, off-target mutations were fully suppressed in rice calli and regenerated plants. However, on-target mutation frequency also decreased compared with that induced by the Cas9 paired nucleases system. Since the gRNA sequence determines specific binding of Cas9 protein–gRNA ribonucleoproteins at the targeted sequence, the on-target mutation frequency of Cas9 paired nickases depends on the design of paired gRNAs. Our results suggest that a combination of gRNAs that can induce mutations at high efficiency with Cas9 nuclease should be used together with Cas9 nickase. Furthermore, we confirmed that a combination of gRNAs containing a one nucleotide (1 nt) mismatch toward the target sequence could not induce mutations when expressed with Cas9 nickase. Our results clearly show the effectiveness of Cas9 paired nickases in delivering on-target specific mutations.

## Introduction

Recently, CRISPR (clustered regularly interspaced short palindromic repeats)/Cas9 (CRISPR-associated protein 9) has been utilized widely as a genome editing tool in various organisms, including bacteria, yeast, animals and plants (Jinek et al. 2012, Cong et al. 2013, Cho et al. 2013, Hwang et al. 2013, Jiang et al. 2013a, Jinek et al. 2013, Mali et al. 2013a). The CRISPR/Cas9 system uses Cas9 protein and a single guide RNA (gRNA). Cas9 is an RNA-guided endonuclease that can cleave double-stranded DNA, with two separated nuclease domains (the RvuC-like domain and HNH motif) each cleaving one of the double strands. Target specificity is governed by the gRNA, which binds directly to a 20 nucleotide (nt) sequence on the target DNA. In the binding site of CRISPR/Cas9, the 20 nt matching the gRNA and the sequence motif 5′-NGG-3′ is located immediately after the 20 nt target DNA, which Cas9 from *Streptococcus pyogenes* recognizes as a protospacer adjacent motif (PAM) sequence (Jinek et al. 2012). The PAM sequence plays an important role in binding to, and breaking, the target DNA (Sternberg et al. 2014).

The target specificity of CRISPR/Cas9 is determined by 20 nt of the gRNA together with the PAM sequence, and is dependent on the number and position of mismatches in the 20 nt gRNA sequence (Cong et al. 2013, Fu et al. 2013, Hsu et al. 2013, Pattanayak et al. 2013). CRISPR/Cas9 tolerates mismatches, especially at the 5′ end of the gRNA sequence, but the seed region of approximately 12 nt proximal to the PAM sequence is crucial for binding to the target DNA (Cong et al. 2013, Fu et al. 2013, Hsu et al. 2013, Pattanyak et al. 2013). In addition, the frequency of off-target mutations varies depending on the expression method of Cas9 and gRNA, and the organism (Fu et al. 2013, Shen et al. 2013, Cho et al. 2014, Smith et al. 2014, Veres et al. 2014). To suppress off-target mutations, Cas9 paired nickases have been utilized in mammalian systems (Mali et al. 2013b, Ran et al. 2013). Cas9 nickase contains a mutation in one of the nuclease domains—the RuvC-like (D10A) or HNH (H840A) domains—existing in Cas9 nuclease (Jinek et al. 2012). Double nicks, i.e. two DNA single-strand breaks (SSBs) induced by Cas9 nickase and the paired gRNAs that bind to opposite DNA strands, result in the induction of a DNA double strand break (DSB). Because Cas9 paired nickases can improve the specificity of the CRISPR/Cas9 system by doubling the recognition site of 20 nt, this method effectively suppresses Cas9 nuclease-induced off-target mutations in mammalian cells (Shen et al. 2013, Cho et al. 2014, Kuscu et al. 2014, Smith et al. 2014, Veres et al. 2014). The result reported for on-target mutation frequency using Cas9 paired nickases varies depending on the type of overhang (5′- or 3′-overhang), and the distance between the paired gRNAs sites (Mali et al. 2013b, Cho et al. 2014, Shen et al. 2013).

Recently, many studies have demonstrated successful CRISPR/Cas9-mediated genome editing and heritable mutations in several plants species, e.g. Arabidopsis (Feng et al. 2013, Jiang et al. 2013a, Li et al. 2013, Jiang et al. 2014), tobacco (Jiang et al. 2013b, Li et al. 2013, Nekrasov et al. 2013), rice (Feng et al. 2013, Miao et al. 2013, Shan et al. 2013, Xu et al. 2014, Zhang et al. 2014, Zhou et al. 2014, Endo et al. 2015, Mikami et al. 2015), wheat (Upadhyay et al. 2013, Wang et al. 2014), *Zea mays* (Svitashev et al. 2015), soybean (Sun et al. 2015), tomato (Brooks et al. 2015, Ito et al. 2015) and populus (Fan et al. 2015). Thus, CRISPR/Cas9 is becoming a powerful tool for editing plant genomes, including those of crops. However, in studies using Cas9 nuclease, off-target mutations were detected in the rice genome, where 1 nt or 2 nt mismatches exist toward on-target sites (Zhang et al. 2014, Endo et al. 2015). Off-target mutations are problematic if the goal is functional analysis of a gene of interest. Furthermore, whole-genome sequencing has not been completed in most plant species, and the risk of off-target mutations is thus unpredictable in many cases. So, establishment of a Cas9 paired nickases-mediated targeted mutagenesis, like that known to suppress off-target mutations in mammals, would also be desirable in plants. In Arabidopsis, reports of successful Cas9 paired nickases-mediated targeted mutagenesis have appeared recently (Fauser et al. 2014, Schiml et al. 2014). However, a comprehensive analysis of Cas9 paired nickases including optimal designs of gRNAs for avoiding off-target mutations has not yet been reported for any plant species.

**Fig. 1:**
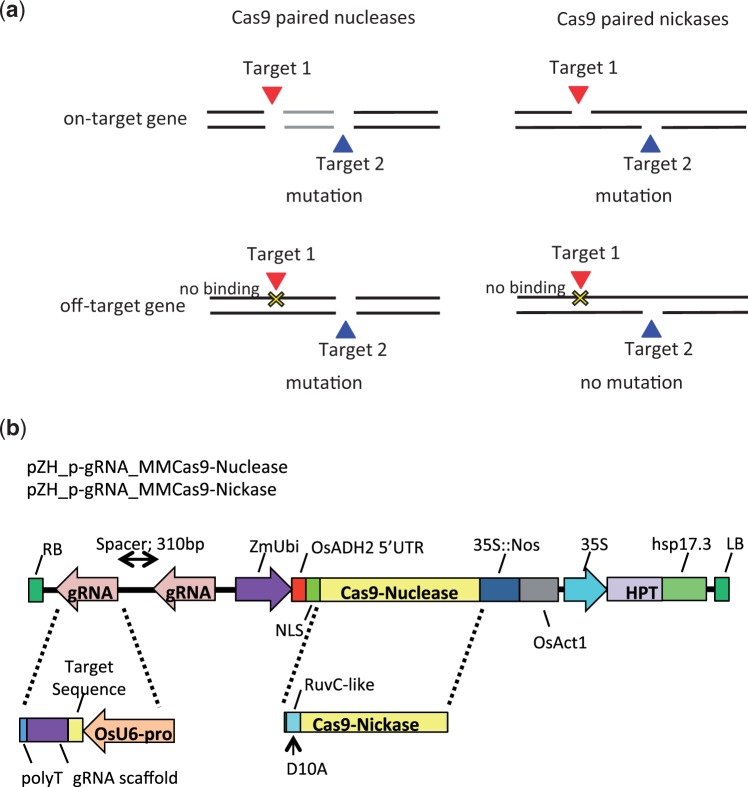
Strategy and vector constructions in this study. (a) Strategy for inducing on- and off-target mutations with Cas9 nuclease or Cas9 nickase. (b) Expression constructions for Cas9 nuclease or Cas9 nickase harboring paired gRNAs expression cassettes.

To verify the utility of Cas9 paired nickases in plants, we conducted our study in rice, because the whole-genome sequence of rice (*Oryza sativa* L. cv. Nipponbare) has been published (International Rice Genome Sequencing Project 2005), and target sites with or without off-target candidates can be selected easily. We first considered the rice *disrupted meiotic cDNA 1 A* (*OsDMC1A*) gene as the ‘on-target’ gene, and the *OsDMC1B* gene—a paralog of *OsDMC1A* with high homology to *OsDMC1A* (Sakane et al. 2008) —as the ‘off-target’ gene, and compared the mutation frequency in both genes upon using Cas9 paired nucleases or Cas9 paired nickases with several paired gRNAs. Next, we designed gRNAs with different numbers of mismatches toward the target sequence on *OsDMC1A.* Paired gRNAs possessing 0, 1 or 2 nt mismatches were expressed with Cas9 nuclease or Cas9 nickase, to explore the effect of different numbers of mismatches on on- and off-target mutation efficiency. Schematic representation of on- and off-target mutations induced by Cas9 nuclease or Cas9 nickase is shown in Fig. 1a.

## Results

### Vector construction and evaluation of mutation frequency

The *S. pyogenes* Cas9 nuclease-coding sequence codon-modified for rice was used throughout this study (MMCas9-Nuclease; Mikami et al. 2015). To design a Cas9 nickase containing a point mutation disrupting the RuvC-like domain (D10A) (Jinek et al. 2012), we constructed MMCas9-Nickase from MMCas9-Nuclease. Regulatory elements of Cas9 nuclease and Cas9 nickase are the promoter, the first exon and first intron sequence of the maize polyubiquitin gene (ZmUbi) (Takimoto et al. 1994), and a double terminator [the Cauliflower mosaic virus (CaMV) 35S terminator (35S), and nopaline synthase terminator from Agrobacterium tumefaciens (Nos)]. The gRNA is driven by the RNA polymerase III promoter from the rice U6-2 small nuclear RNA gene (OsU6) (Feng et al. 2013, Mikami et al. 2015). For paired gRNAs, two gRNAs expression constructs—each recognition sequence separated by 310bp—were arranged in tandem and cloned into the all-in-one vector harboring the Cas9 nuclease or Cas9 nickase expression constructs, respectively (pZH_p-gRNA_MMCas9-Nuclease and pZH_p-gRNA_MMCas9-Nickase; Fig. 1b).

One month after transformation, genomic DNA was extracted from independent transgenic calli and subjected to CAPS (cleaved amplified polymorphic sequences) analysis or CEL I analysis to detect mutations at the target sequence. We evaluated the mutation frequency of Cas9/gRNA-transformed calli using two criteria (Supplementary Fig. S1): (i) we defined the number of calli with CRISPR/Cas9-mediated mutations per total number of Cas9/gRNA-transformed calli as the ‘ratio of calli with mutations (RC)’; and (ii) PCR products of clonally propagated calli were cloned, and the ratio of mutated to non-mutated sequence was evaluated by DNA sequencing. Mutation frequencies (MFs) in independent calli were determined, and the average mutation frequency (AMF) in the top three highly mutated calli was determined (Supplementary Fig. S1).

### Evaluation of on- and off-target mutations using Cas9 paired nucleases

**Fig. 2:**
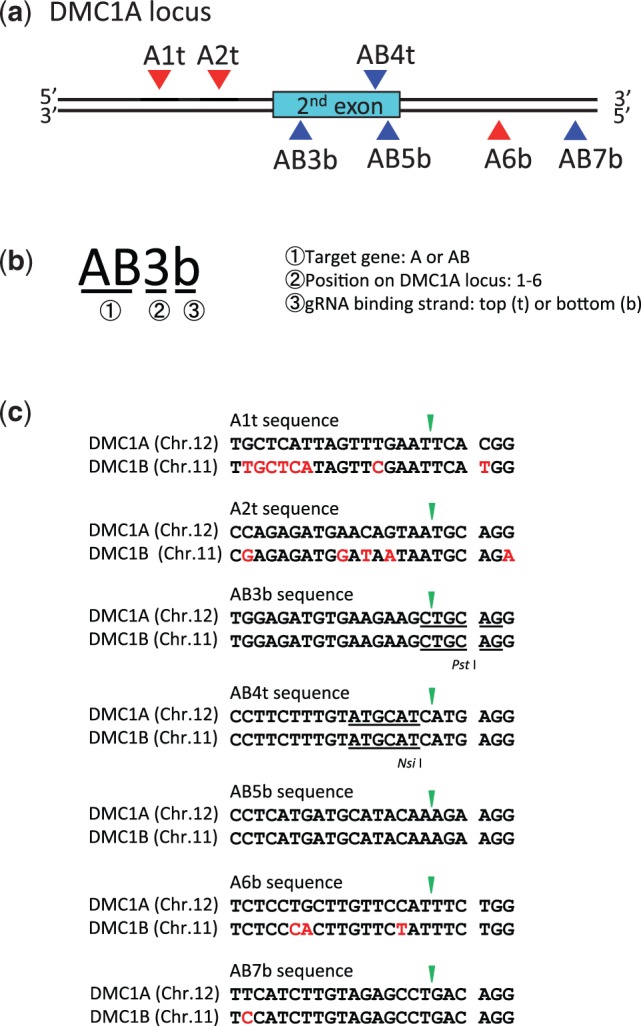
Target sequences on *OsDMC1* genes. (a) Target sites on *OsDMC1A.* Blue arrowhead, gRNA recognizes the target site on *OsDMC1A* and *OsDMC1B*; red arrowhead, gRNA recognizes the target site on *OsDMC1A*; cyan bar, second exon of *OsDMC1A.* (b) Naming rules for gRNAs on *OsDMC1A.* (c) Homology and mismatch of target sequences in *OsDMC1A* and *OsDMC1B.* Mismatches to the target sequence on *OsDMC1* are shown in red. The green arrowhead indicates the expected cleavage site. Restriction enzyme recognition sequences are underlined.

**Fig. 3:**
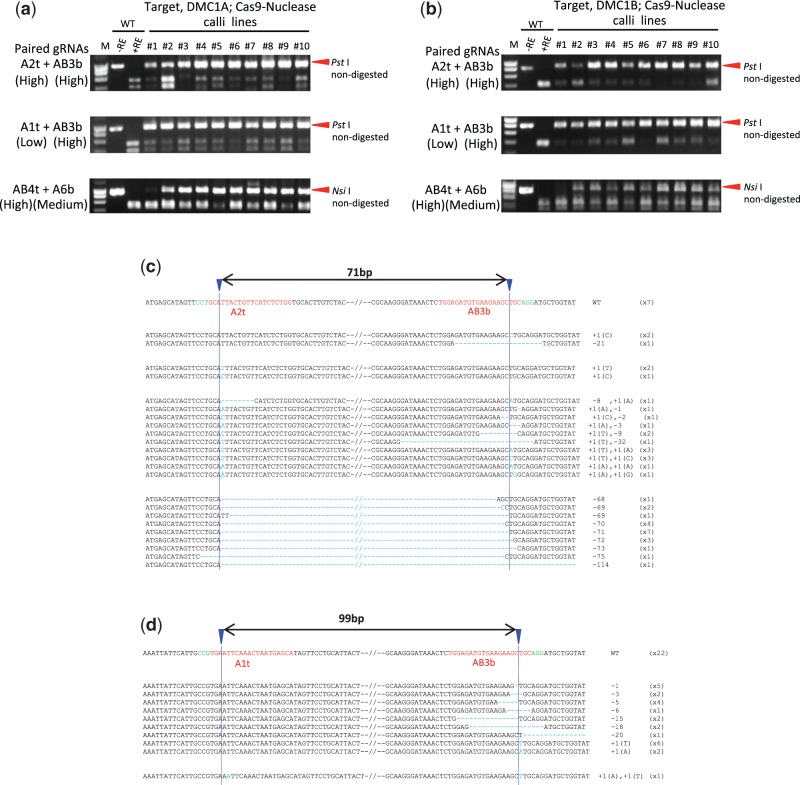
Mutations induced by Cas9 paired nucleases. (a) CAPS analysis of the on-target gene, *DMC1A.* M, molecular weight marker; –RE, PCR product without restriction enzyme reaction; +RE, restriction enzyme-digested PCR product; WT, non-transformed calli; #1–10, independent transformed calli lines. (b) CAPS analysis of the off-target gene, *DMC1B.* (c) Mutation variations on A2t and/or AB3b sites using Cas9 nuclease. The wild-type sequence is shown at the top with the PAM sequence in green, and the 20 nt target sequence in red. The blue arrowhead indicates the expected cleavage site. Dashes, deleted bases. The net changes in length are shown to the right of each sequence (+, insertion; –, deletion). The number of clones representing each mutant allele is shown in brackets. (d) Total mutation variations on A1t and/or AB3b sites using Cas9 nuclease.

Three *OsDMC1A*-specific gRNAs (A1t, A2t and A6b) were designed, and four other gRNAs (AB3b, AB4t, AB5b and AB7b) were designed based on consensus sequences of *OsDMC1A* and *OsDMC1B* (Fig. 2a). Naming rules for gRNAs on OsDMC1A(italic type) are shown in Fig. 2b. We analyzed RC scores and MF values in calli expressing a single gRNA and Cas9 nuclease, and evaluated the performance of each gRNA based on the MF (high, medium and low in Supplementary Table S1). Next, six sets of gRNAs (A1t + AB3b, A2t + AB3b, AB4t + AB3b, AB4t + AB5b, AB4t + A6b and AB4t + AB7b; Table 1) were selected for the construction of pZH_p-gRNA_MMCas9-Nuclease vectors. Mutations generated in transgenic calli were subjected to CAPS analysis since potential mutation sites of AB4t and AB3b gRNAs contained *Nsi* I and *Pst* I recognition sequences, respectively (Fig. 2c). Because the MF of both the *OsDMC1A* and *OsDMC1B* genes using a single AB4t gRNA or AB3b gRNA was extremely high with Cas9 nuclease (Supplementary Table S1), RC scores of both genes were approximately 100% in six paired gRNAs sets with Cas9 nuclease. AMF values of *OsDMC1A* were almost the same regardless of whether paired gRNAs or a single gRNA were used (Figs. 3a, b; Table 1; Supplementary Table S1). In Cas9 paired nucleases using the A2t + AB3b set, the AMF value of *OsDMC1A* was 85.4% and classification of MF and variations was as follows: DNA with mutations in one of the two target sites was 6.2% at each site; DNA with mutations in both target sites was 31.2%; and deletion between two target sites was 41.6% (Fig. 3c; Tables 1, 2). Using the A1t + AB3b set, the AMF value of *OsDMC1A* was 54.1%. However, most mutations were induced in the AB3b site (52%), and deletions between the A1t + AB3b sites (99 bp) were not detected (Fig. 3d; Tables 1, 2). These results indicate that a combination of high performance gRNAs is necessary to direct deletions between the two target sites in Cas9 paired nucleases (Table 1; Supplementary Table S1). Furthermore, a deletion of approximately 71 bp between the two target sites, A2t and AB3b, was found in 13 out of 31 regenerated plants (41%), and mono- or bi-allelic mutated plants with various mutations were obtained (Table 3).

**Table 1:** Mutation rates in pZH_p-gRNA_MMCas9-Nuclease- or pZH_p-gRNA_MMCas9-Nickase-transformed calli

Paired gRNAs(MF rank*^a^*)		Target gene	Overhang	Distance of paired gRNAs (bp)	Cas9 paired nucleases		Cas9 paired nickases	
					RC*^b^*	AMF (%)*^c^*	RC*^b^*	AMF (%)*^c^*
A1t	AB3b	DMC1A	5′	99	24/24	54.1	1/24	ND
(Low)	(High)	DMC1B	–	–	23/24	70.4	0/24	–

A2t	AB3b	DMC1A	5′	71	24/24	85.4	23/24	56.2
(High)	(High)	DMC1B	–	–	24/24	75	0/24	–

A2t-1	AB3b-1	DMC1A	5′	71			24/24	ND

A2t-2	AB3b-2	DMC1A	5′	71			0/24	–

AB4t	AB3b	DMC1A	3′	37	24/24^d^	84	2/24*^d^*	ND
(High)	(High)	DMC1B	3′	37	24/24^d^	79.4	1/24*^d^*	ND

AB4t	AB5b	DMC1A	5′	11	23/24	47.9	14/24	18.7
(High)	(Medium)	DMC1B	5′	11	22/24	25.0	13/24	10.4

AB4t	A6b	DMC1A	5′	66	23/24	64.5	20/24	40.4
(High)	(Medium)	DMC1B	–	–	23/24	43.3	0/24	–

AB4t	AB7b	DMC1A	5′	167	23/24	43.1	1/24	ND
(High)	(Low)	DMC1B	–	–	23/24	37.6	0/24	–

B1t	BA1b	CDKB2	5′	30	20/24	41.6	24/24	22.7
		CDKA2	–	–	20/24	33.3	0/24	–
(Medium)	(Medium)	CDKB1	–	–	13/24	8.3	0/24	–
		CDKA1	–	–	5/24	2.0	0/24	–

B2t	BA1b	CDKB2	5′	127	18/24	40.9	20/24	14.5
		CDKA2	–	–	18/24	29.1	0/24	–
(Medium)	(Medium)	CDKB1	–	–	12/24	4.5	0/24	–
		CDKA1	–	–	2/24	2.0	0/24	–

The total number of sequenced clones is approximately 48 (16 clones × 3 lines).

*^a^* The rank of mutation frequency (MF) using single gRNA and Cas9 nuclease (Supplementary Table S1).

*^b^* Ratio of calli with mutations (RC) by CAPS analysis of gRNA which recognizes on- and off-target sites (AB3b, AB4t and BA1b).

*^c^* Average mutation frequency (AMF) of three lines (Supplementary Fig. S1).

*^d^* CAPS analysis of AB3b site.

**Table 2:** Mutation variations of pZH_p-gRNA_MMCas9-Nuclease transformed calli in the *DMC1A* gene

Paired gRNAs		AB4t+AB3b	A2t+AB3b	A1t+AB3b	AB4t+A6b
**Distance of paired gRNAs**		**37bp**	**71bp**	**99bp**	**66bp**

	Recognition strand				
		
Total number of mutated clones	Top-strand mutation	2	3	0	12
		(4.5%)	(6.2%)	(0%)	(25%)
	
	Bottom-strand mutation	6	3	25	1
		(13.6%)	(6.2%)	(52%)	(2%)
	
	Top- and Bottom-strand mutations	7	15	1	7
		(15.9%)	(31.2%)	(2.2%)	(14.5%)
	
	Deletion between two target sites	22	20	0	9
		(50%)	(41.6%)	(0%)	(18.7%)
	
Number of no-mutated clones		7	7	22	17
		(15.9%)	(14.5%)	(45.8%)	(35.4%)
Total number of sequenced clones		44	48	48	48

**Table 3:** Mutation variations of transgenic plants regenerated from pZH_p-gRNA_MMCas9-Nuclease- or pZH_p-gRNA_MMCas9-Nickase-transformed calli

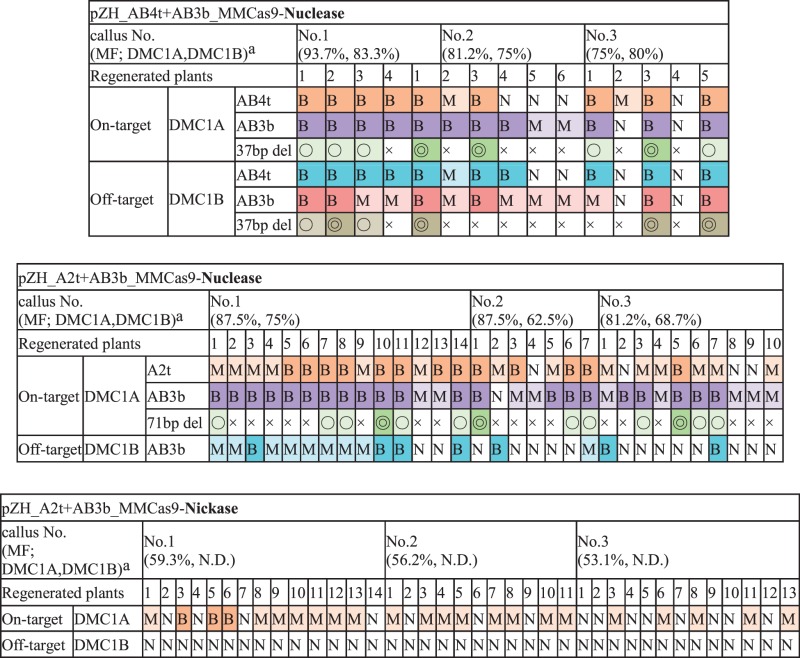

*^a^*Callus No. is the order of top three high mutated calli in total 24 lines.

MF: Mutation frequency

B, bi-allelic mutation; M, mono-allelic mutation; N, non-mutation

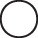
, deletion between two target sites on mono-allelic.

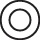
, deletion between two target sites on bi-allelic

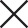
, no- deletion between two target sites

### On-target mutations induced by Cas9 paired nickases

**Fig. 4:**
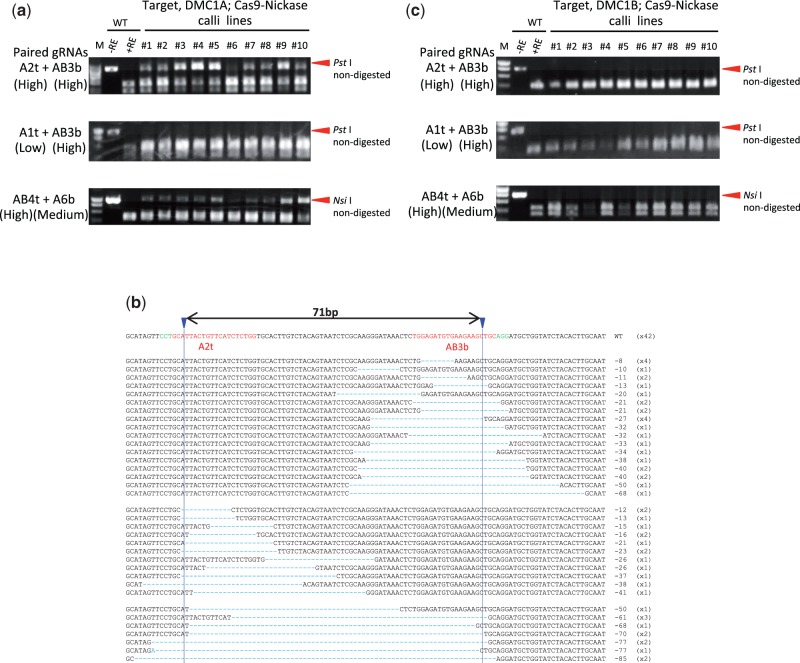
Mutations induced by Cas9 paired nickases. (a) CAPS analysis of the on-target gene, *DMC1A.* M, molecular weight marker; –RE, PCR product without restriction enzyme reaction; +RE, restriction enzyme-digested PCR product; WT, non-transformed calli; #1–10, independent transformed calli lines. (b) Total mutation variations on A2t and/or AB3b sites using Cas9 paired nickases. The wild-type sequence is shown at the top with the PAM sequence in green, and the 20 nt target sequence in red. The blue arrowhead indicates the expected cleavage site. Dashes indicate deleted bases. The net changes in length are shown to the right of each sequence (–, deletion). The number of clones representing each mutant allele is shown in brackets. (c) CAPS analysis of the off-target gene, *DMC1B*.

Because Cas9 nickase produces a nick on each DNA strand, a pair of nicks must be induced simultaneously with high efficiency to ensure efficient targeted mutagenesis. We thus determined the Cas9 paired nickases-mediated MF using paired gRNAs with various performances (Supplementary Table S1). The RC scores for the on-target gene, *OsDMC1A*, with the A2t + AB3b, AB4t + AB5b and AB4t + A6b gRNA sets were 23/24 (95.8%), 14/24 (58.3%) and 20/24 (83.3%), while those with the A1t + AB3b, AB4t + AB3b and AB4t + AB7b sets were 1/24 (4.1%), 2/24 (8.3%) and 1/24 (4.1%), respectively (Fig. 4a; Table 1). This result suggested that a combination of high performance gRNAs was necessary for efficient targeted mutagenesis using Cas9 paired nickases. When set A2t + AB3b was used with Cas9 nickase, the AMF value of *OsDMC1A* was 56.2%, and variations of on-target mutations were deletions detected around either the A2t (14.5%) or AB3b (29.1%) site, or between the A2t and AB3b sites (12.5%) (Fig. 4b; Table 1). In the A2t + AB3b set, the AMF value of *OsDMC1A* using Cas9 paired nickases decreased compared with that obtained with Cas9 paired nucleases (Table 1). A similar result was also seen when several paired gRNAs sets were used on the *OsDMC1A* and rice *Cyclin Dependent Kinase* (*OsCDK*) genes (Table 1; Supplementary Fig. S2). The number of bi-allelic plants with mutations in the on-target gene produced by Cas9 paired nucleases was higher than that produced by Cas9 paired nickases (Table 3; Supplementary Table S2). In conclusion, when on-target mutation frequency takes priority, the use of Cas9 paired nucleases is superior to that of Cas9 paired nickases.

### Off-target mutations are suppressed by Cas9 paired nickases in rice

The above results revealed that higher MF of the on-target gene, *OsDMC1A*, was achieved using Cas9 paired nucleases than with Cas9 paired nickases. However, Cas9 nuclease also induced mutations in the off-target gene, *OsDMC1B*, because one of the paired gRNAs (AB3b, AB4t, AB5b and AB7b) recognized the common sequence between *OsDMC1A* and *OsDMC1B* (Fig. 3b; Table 1). In the case of Cas9 paired nickases, double nicks must be induced efficiently to effect mutation, and mismatches in a single gRNA might suppress off-target mutations. When Cas9 nickase was expressed with A2t + AB3b or AB4t + A6b sets (Fig. 2c), off-target mutations on *OsDMC1B* were below the limit of detection possible with CAPS analysis (Fig. 4c). This result means that 5 nt and 3 nt mismatches in the A2t and A6b sites are enough to suppress off-target mutations even if the other gRNAs match the *OsDMC1B* sequence perfectly. This reduction in off-target mutations using Cas9 paired nickases was further confirmed in an experiment in which *OsCDK* family genes were used as on- and off-target genes. Mutations in off-target candidate genes (*OsCDKA2*, *OsCDKB1* and *OsCDKA1*; Endo et al. 2015) were reduced dramatically when Cas9 paired nickases were used (Table 1; Supplementary Fig. S2). As expected, mutations were induced in both *OsDMC1A* and *OsDMC1B* when paired gRNAs, which recognize a common sequence in these genes, were expressed with Cas9 nickase (AB4t + AB3b and AB4t + AB5b sets in Table 1 and Fig. 2c). In the case of regenerated plants obtained from transgenic calli generated using Cas9 nickase and the A2t + AB3b set, the ratio of regenerated plants with on-target mutations was 60.5% (23/38), and no off-target mutations were detected in any of the regenerated plants (Table 3). Similar results were obtained when *OsCDK* family genes were used as on- and off- target genes (Supplementary Table S2). Even if one of the gRNAs recognizes a consensus sequence in the on- and off-target genes, off-target mutation can be avoided by using Cas9 nickase and the other gRNAs, which recognizes the on-target gene specifically.

### Effect of number of mismatches on the target sequence using Cas9 paired nickases

**Fig. 5:**
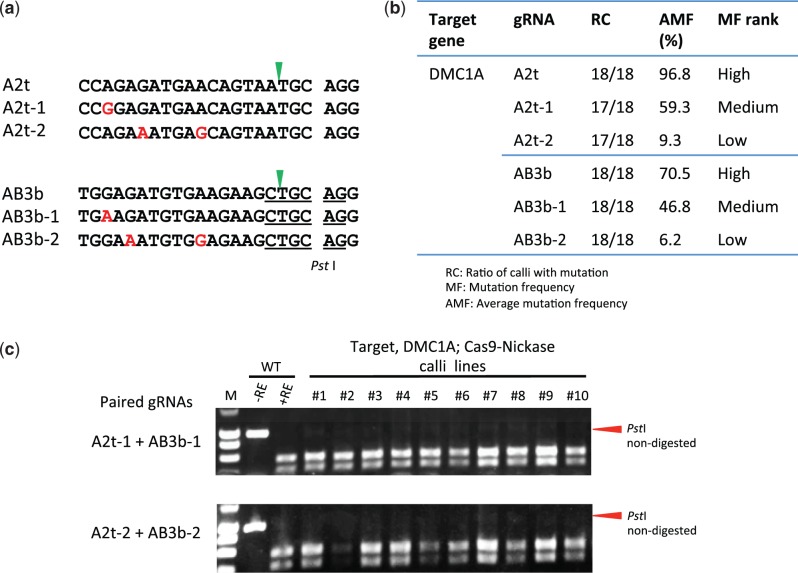
Mutations induced by Cas9 nickase and paired gRNAs with mismatch on *OsDMC1A.* (a) Homology and mismatch of target sequences in *OsDMC1A.* Mismatches to the target sequence on *OsDMC1A* are shown in red. The green arrowhead indicates the expected cleavage site. The *Pst* I restriction enzyme recognition sequence is underlined. (b) Mutation frequency using single gRNA and Cas9 nuclease. (c) CAPS analysis of *OsDMC1A.* M, molecular weight marker; –RE, PCR product without restriction enzyme reaction; +RE, restriction enzyme-digested PCR product; WT, non-transformed calli; #1–10, independent transformed calli lines.

To evaluate the accuracy of Cas9 paired nickases in detail, all experimental conditions except the number of mismatches on the gRNAs were kept the same. Thus, we designed gRNAs containing 0, 1 or 2 nt mismatches on the *OsDMC1A* sequence (A2t-1, A2t-2, AB3b-1 and AB3b-2; Fig. 5a) and considered *OsDMC1A* as the on- or off-target gene depending on the situation. When these gRNAs were transformed together with Cas9 nuclease independently, the AMF values were 96.8% (A2t), 59.3% (A2t-1), 9.3% (A2t-2), 70.5% (AB3b), 46.8% (AB3b-1) and 6.2% (AB3b-2), respectively (Fig. 5b; Supplementary Table S1). Thus, an increased number of mismatches on the gRNA expressed with Cas9 nuclease reduces the off-target mutation frequency but not enough to suppress it completely. Next, combinations of gRNAs that were perfectly matched (A2t + AB3b), 1 nt mismatched (A2t-1 + AB3b-1) or 2 nt mismatched (A2t-2 + AB3b-2) toward the *OsDMC1A* sequence were expressed with Cas9 nickase. In these cases, both RC scores and AMF values were effectively 0% (Fig. 5c; Table 1). These results suggest that if two gRNAs each recognize an off-target site individually, the combination of these gRNAs used with Cas9 nickase can drastically suppress off-target mutations.

## Discussion

Previously, we reported the knockout of multigenes by taking advantage of off-target mutations in the CRISPR/Cas9 system (Endo et al. 2015). Off-target mutation is useful in some cases, especially when multiple homologous genes are the targets of disruption. However, off-target mutation is considered troublesome in most cases, and utilization of Cas9 paired nickases has been reported as a promising approach to preventing off-target mutation in mammals (Cho et al. 2013, Ran et al. 2013, Shen et al. 2013). Recently, Cas9 paired nickases-mediated mutagenesis was reported in Arabidopsis, although the frequency of on-target mutation was decreased (Fauser et al. 2014, Schiml et al. 2014). However, the applicability of Cas9 paired nickases to suppressing off-target mutations in plants has not yet been elucidated in detail.

Here, we first considered *OsDMC1A* and *OsCDKB2* as ‘on-target’ genes, and their paralogs *OsDMC1B*, *OsCDKA2*, *OsCDKB1* and *OsCDKA1* as ‘off-target’ genes, in order to compare the ratio of on-target and off-target mutations between Cas9 paired nucleases and Cas9 paired nickases. Even when the target sequence of one member of a paired gRNA perfectly matched both on- and off-target genes, off-target mutation was not induced when such gRNAs were expressed with Cas9 nickase and the other gRNA of the pair, which had mismatches of >3 nt toward the off-target gene (Fig. 2c). To investigate the accuracy of Cas9 paired nickases in more detail, we next designed gRNAs with different numbers of mismatches toward *OsDMC1A* (Fig. 5a), and compared mutation efficiency at the same target site. When gRNAs with 1 or 2 nt mismatches were expressed with Cas9 nuclease, mutations were induced in *OsDMC1A* to some degree (Fig. 5b), although the use of these off-target inducible gRNAs with Cas9 nickases failed to induce mutations on *OsDMC1A* (Figs. 4a, 5c; Table 1). This result verified that Cas9 paired nickases can effectively suppress off-target mutations. However, the number and position of mismatches in the 20 nt target sequence are the most crucial factors for binding of the Cas9–gRNA complex to the target sequence (Cong et al. 2013, Fu et al. 2013, Hsu et al. 2013, Pattanayak et al. 2013). In Cas9 paired nickases, further detailed analysis of the number and position of mismatches in the 40 nt target sequences may be needed to reduce the risk of off-target mutations.

As mentioned above, decreased frequency of on-target mutation is one of the concerns regarding the use of Cas9 paired nickases. In our results, differences in on-target MF between Cas9 paired nucleases and Cas9 paired nickases varied depending on the gRNA sequences used. The smallest difference was between the 41.6% (nuclease) and 22.7% (nickase) observed in paired gRNAs B1t and BA1b on the *OsCDKB2* gene (Table 1). Meanwhile, the largest difference was between the 84% (nuclease) and 0% (nickase) with paired gRNAs AB4t and AB3b on the *OsDMC1A* gene (Table 1; Figs. 3a, 4a; Supplementary Fig. S2). In the case of Cas9 paired nickases, both nicks must be induced efficiently to create a DSB. Thus, before using a pair of gRNAs with Cas9 nickase, it is recommended to check whether each of the gRNAs can induce mutations efficiently when expressed with Cas9 nuclease.

In human cells, it was reported that the 3′-overhang structure made by Cas9 paired nickases induced fewer mutations than a 5′-overhang structure (Mali et al. 2013b, Cho et al. 2014). In our study, a 5′-overhang structure was formed in six paired gRNAs sets, and mutations were induced efficiently when both gRNAs acted in concert with Cas9 nuclease (Table 1). In contrast, a 3′-overhang structure was formed in one paired gRNAs set (AB4t + AB3b) and mutations were barely induced at all using Cas9 nickase even though both members of each paired gRNAs set acted well together with Cas9 nuclease and the distance between the two nicks was relatively short (37 bp) (Table 1). This result might suggest that a 3′-overhang structure also induces fewer mutations compared with 5′-overhangs in rice. We speculate that this difference might be due to the resection step during the DNA repair process. There are at least two repair pathways for DSB repair: non-homologous end-joining (NHEJ), a simple error-prone end-joining mechanism that is a major pathway of DSB repair; and homologous recombination (HR), an error-free DNA repair mechanism that uses homologous DNA sequences such as sister chromatids as templates (Heyer et al. 2010). The initial steps of HR-mediated DNA DSB repair are: (i) processing of DSBs to create a 3′-overhang structure; (ii) polymerization of Rad51 on this single-stranded DNA; (iii) a Rad51-DNA filament-directed homology search; (iv) strand invasion into undamaged homologous template duplex DNA; and (v) formation of a D-loop structure that can be processed further by synthesis-dependent strand annealing (SDSA) during HR (Heyer et al. 2010, Symington and Gautier 2011). The 3′-overhang structure created by Cas9 paired nickases might trigger HR by helping the initial resection step of HR. Because HR repair using a sister chromatid as a template is error-free, a few mutations might be induced using Cas9 paired nickases to create the 3′-overhang structure. Clearly, further study is needed to investigate the effect of the DSB end structure on MF in plants.

Off-target MF was seen to vary depending on the amount of Cas9 mRNA and gRNA injected into human cells (Fu et al. 2013, Hsu et al. 2013). Similar concerns are not negligible in CRISPR/Cas9-mediated targeted mutagenesis in plants. Recently, successful DNA-free plant genome editing using Cas9 protein–gRNA ribonucleoproteins (RNPs) injected into protoplasts has been reported (Woo et al. 2015). In this method, the amount of Cas9 and gRNA used for injection can be regulated, and nuclease activity does not persist for long due to digestion of the Cas9 protein and the gRNA. This method thus seems to have advantages of decreasing off-target mutation in addition to being exempt from GMO regulation.

In conclusion, the specific features of the Cas9 paired nickases system in plants can be summarized as follows: (i) off-target mutations are suppressed significantly using Cas9 paired nickases, as also reported in human cells (Cho et al. 2013, Ran et al. 2013, Shen et al. 2013); (ii) MF using Cas9 paired nickases tends to drop compared with that obtained using Cas9 nuclease (Cho et al. 2013, Fauser et al. 2014, Schiml et al. 2014); (iii) a combination of high performance paired gRNAs is needed for efficient on-target mutation using Cas9 paired nickases; and (iv) depending on the structure of overhangs, 5′- or 3′-overhangs, and the distance between nicks, MF can be altered.

In the case of plant species for which the whole-genome sequence is available, we can select gRNAs without off-target candidates or confirm the presence or absence of mutations in off-target candidate sites. However, such plant species are still few in number, and polymorphisms exist among cultivars. Because the use of Cas9 paired nickases significantly suppresses off-target mutations, mutagenesis mediated by Cas9 paired nickases will be exceedingly effective for targeted mutagenesis of a wide range of plant species. Accumulation of data involved in complex formation of Cas9, gRNA and genomic DNA; improvements in the expression system of Cas9 and gRNA; and understanding of the DNA repair mechanism will further enhance an efficient genome editing system free from off-target mutations in plants.

## Materials and Methods

### Construction of gRNA/Cas9 all-in-one vectors

The vectors in this study are based on our previously described Cas9 cloning vectors (pZH_OsU6gRNA_MMCas9; Mikami et al. 2015). The pZH_OsU6gRNA_MMCas9-Nickase vector was constructed by PCR-based site-directed mutagenesis. The pZH_p-gRNA_MMCas9-Nuclease and pZH_p-gRNA_MMCas9-Nickase vectors were made as follows. (i) pU6gRNA-oligo (Mikami et al. 2015) has two *Bbs* I sites between the OsU6 promoter and the gRNA scaffold sequence. These vectors were both linearized using *Bbs* I, and the 20 nt annealed oligonucleotides were ligated into these restriction enzyme recognition sites. (ii) To connect two gRNA expression cassettes, one gRNA expression cassette (OsU6pro::gRNA::polyT) was eliminated from pU6gRNA-oligo by using *Pvu* II and *Asc* I, and inserted between the *Eco*RV and *Asc* I sites of the other pU6gRNA-oligo to complete p2x_OsU6gRNA. (iii) Connected gRNA expression cassettes were excised from the p2x_OsU6gRNA vector using *Asc* I and *Pvu* II digestion, and inserted into pZH_OsU6gRNA_MMCas9 and pZH_OsU6gRNA _MMCas9-Nickase vectors using *Pml* I and *Asc* I sites to complete the pZH_p-gRNA_MMCas9-Nuclease and pZH_p-gRNA_MMCas9-Nickase vectors.

### Transformation of rice with Cas9 and gRNA expression constructs

*Agrobacterium*-mediated transformation of rice (*Oryza sativa* L. cv. Nipponbare) using scutellum-derived calli was performed as described previously (Toki 1997, Toki, et al. 2006). One-month-cultured rice calli were infected by *Agrobacterium* carrying the pZH_p-gRNA_MMCas9-Nuclease or pZH_p-gRNA_MMCas9-Nickase vectors. After 3 days of co-cultivation, infected calli were transferred to fresh callus induction medium (CIM) (Toki 1997) containing 50 mg l^–1^ hygromycin B (Wako Pure Chemicals) and 25 mg l^–1^ meropenem (Wako) to remove *Agrobacterium.* Transgenic calli were selected on hygromycin-containing medium for 3 weeks. Proliferating calli were then transferred to fresh CIM without meropenem and cultured for 1 week. After a total of 4 weeks of selection, transgenic calli of pZH_p-gRNA_MMCas9-Nuclease or pZH_p-gRNA_MMCas9-Nickase were used for analysis of mutation frequency.

### CAPS analysis and CEL I analysis

Genomic DNA was extracted from calli or regenerated plants using an Agencourt Chloropure Kit (Beckman Coulter), and target loci were amplified using the primers listed in Supplementary Table S3. PCR products were subjected to restriction enzyme or CEL I nuclease reaction and analyzed by agarose gel electrophoresis.

### Sequencing analysis

PCR products used for CAPS analysis or CEL I analysis were cloned into pCR-BluntII-TOPO (Invitrogen) and subjected to sequence analysis using an ABI3130 sequencer (Applied Biosystems).

## Supplementary data

Supplementary data are available at PCP online.

## Funding

This research was supported by the Ministry of Agriculture, Forestry and Fisheries of Japan [Genomics for Agricultural Innovation grant PGE1001]; the NIAS Strategic Research Fund; the Council for Science, Technology and Innovation (CSTI); Cross-ministerial Strategic Innovation Promotion Program (SIP); ‘Technologies for creating next-generation agriculture, forestry and fisheries’ [funding agency: Bio-oriented Technology Research Advancement Institution, NARO].

### Disclosures

The authors have no conflicts of interest to declare.

## Supplementary Material

Supplementary Data

## Abbreviations

AMF: average mutation frequency
CAPS: cleaved amplified polymorphic sequences
Cas9: CRISPR-associated protein 9
CDK: cyclin-dependent kinase
CRISPR: clustered regularly interspaced short palindromic repeat
DMC1: disrupted meiotic cDNA 1
DSB: DNA double-strand break
gRNA: guide RNA
HR: homologous recombination
MF: mutation frequency
PAM: protospacer adjacent motif
RC: ratio of calli with mutations
SSB: DNA single-strand break, NHEJ, non-homologous end-joining
