# Supplementary material for: Precision Targeted Mutagenesis via Cas9 Paired Nickases in Rice
Source: Plant Cell Physiol. 2016 Mar 2;57(5):1058–68. doi: 10.1093/pcp/pcw049 (PMC4867050; doi:10.1093/pcp/pcw049)
Supplement: Supplementary Data [file supp_57_5_1058__index.html]

Precision targeted mutagenesis via Cas9 paired nickases in rice — Precision Targeted Mutagenesis via Cas9 Paired Nickases in Rice — Precision Targeted Mutagenesis via Cas9 Paired Nickases in Rice — Supplementary Data 

# Precision Targeted Mutagenesis via Cas9 Paired Nickases in Rice

## Supplementary Data

files

- Supplementary Data - pdf file
