# Supplementary material for: Precision Targeted Mutagenesis via Cas9 Paired Nickases in Rice
Source: Plant Cell Physiol. 2016 Mar 2;57(5):1058–68. doi: 10.1093/pcp/pcw049 (PMC4867050; doi:10.1093/pcp/pcw049)
Supplement: Supplementary Data [file supp_pcw049_pcp-2016-e-00002-File003.pdf]

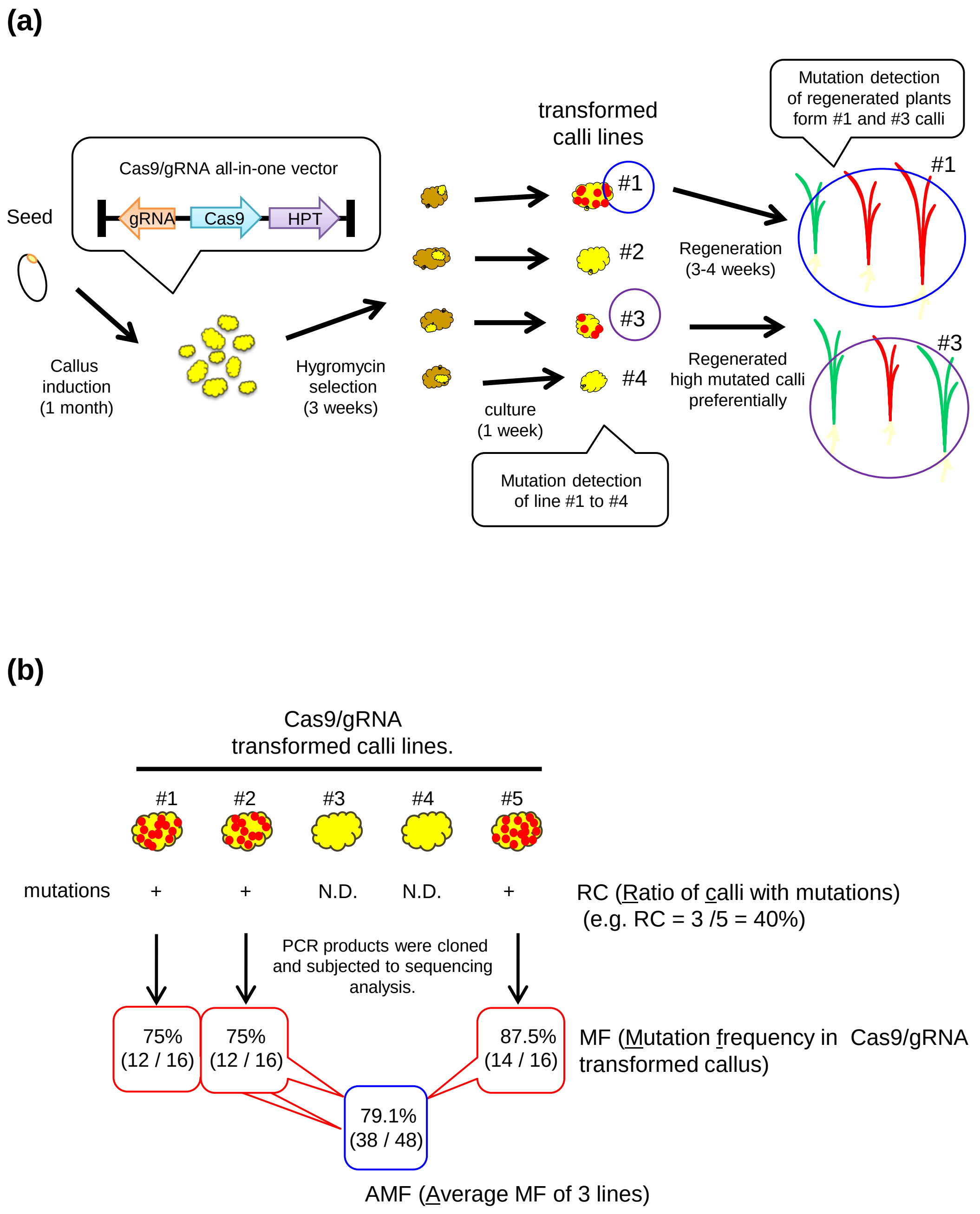

Figure S1. Schematic representation of the CRISPR/Cas9-mediated target mutagenesis employed in this study. (a) Process of transformation and rules of numbering. (b) Evaluation mutation frequency.

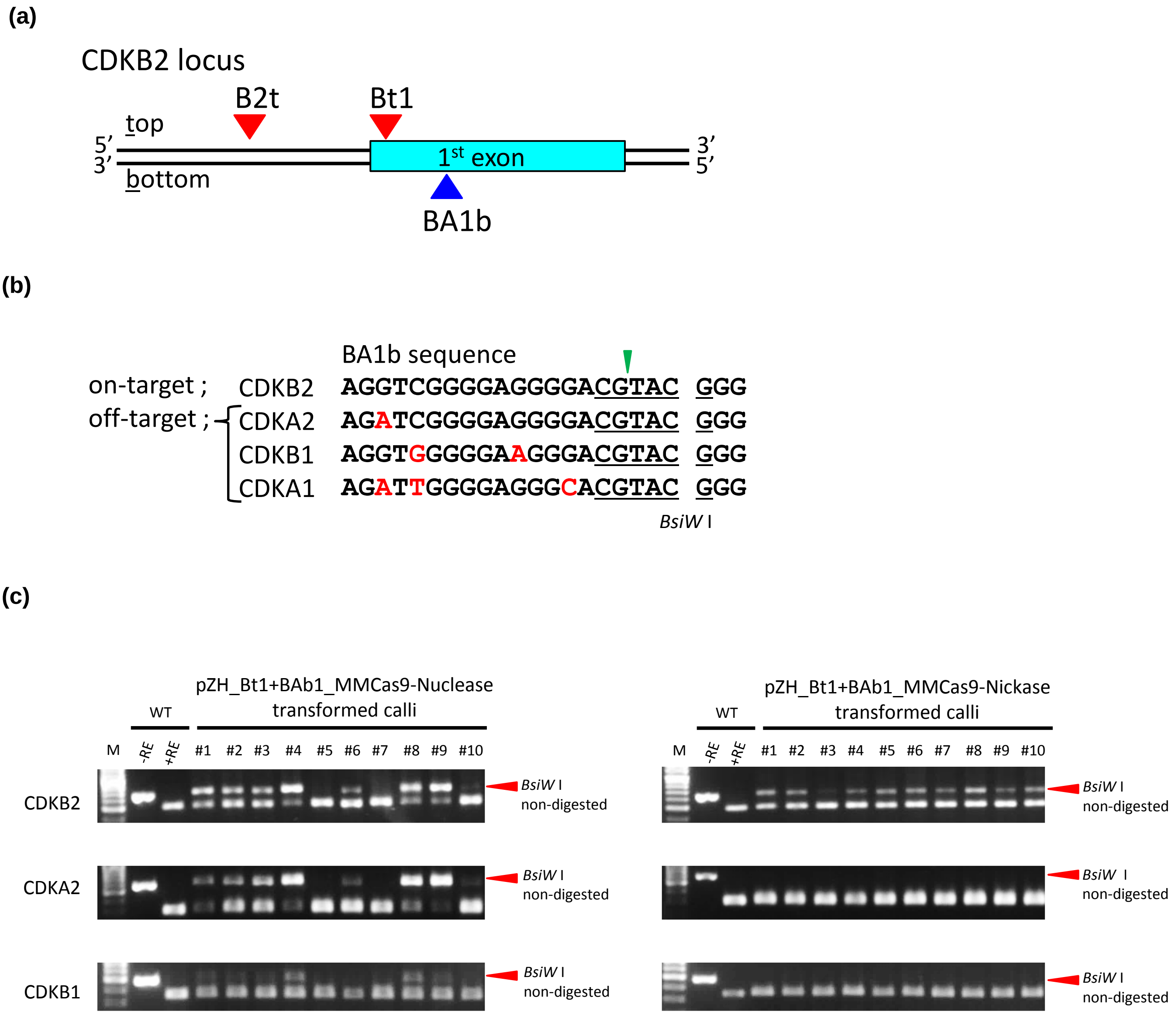

Figure S2. Comparison of Cas9 paired nucleases- and Cas9 paired nickases–mediated targeted mutagenesis on *CDK* gene in rice calli.

(a) Target sites on *CDKB2* gene. Blue arrowhead, gRNA recognizes the target site on *CDKB2*, *CDKA2*, *CDKB1* and *CDKA1* genes; Red arrowhead, gRNA recognizes the target site on *CDKB2* gene. (b) Homology and mismatch of target sequences in *CDK* gene. Mismatches to the target sequence on *CDKB2* gene are shown in red. The green arrowhead indicates the expected cleavage site. The under-line indicates the recognition enzyme sequences.

Table S1. Mutation rates in single gRNA and Cas9 nuclease transformed calli.

	Recognition of gRNA	gRNA name	Target gene	RC†	MF(%) ††		MF rank
					No.1	No.2	
DMC1 genes	on- and off-target sites	AB3b*	DMC1A	18/18 (100)	100	93.7	High
			DMC1B	18/18 (100)	100	94.4	
		AB4t*	DMC1A	18/18 (100)	62.5	55	High
			DMC1B	18/18 (100)	68.7	66.6	
		AB5b**	DMC1A	18/18 (100)	25	25	Medium
			DMC1B	18/18 (100)	25	12.5	
		AB7b**	DMC1A	3/18 (16.6)	18.7	12.5	Low
			DMC1B	3/18 (16.6)	18.7	9.6	
	on-target site	A1t**	DMC1A	2/18 (11.1)	8.3	3.2	Low
			DMC1B	0/18 (0)	-	-	
		A2t**	DMC1A	18/18 (100)	75	66.6	High
			DMC1B	0/18 (0)	-	-	
		A6b**	DMC1A	8/18 (44.4)	30	27.2	Medium
			DMC1B	0/18 (0)	-	-	
CDK genes	on-and off-target sites	BAb1*	CDKB2	18/18 (100)	58.3	50	Medium
			CDKA2	16/18 (88.8)	45.8	34.7	
			CDKB1	14/18 (77.7)	16.6	12.5	
			CDKA1	6/18 (33.3)	4.1	4.1	
	on-target site	B1t**	CDKB2	18/18 (100)	54.5	40	Medium
		B2t**	CDKB2	18/18 (100)	33.3	33.3	Medium

* Mutations were detected by CAPS analysis.

** Mutations were detected by CEL I analysis.

† RC is defined as 'Ratio of calli with mutations'.

† † MF is defined as 'mutation frequency of Cas9/gRNA transformed callus'.

Table S2. Summary of mutation frequency using Cas9 paired nucleases and Cas9 paired nickases in regenerated plants.

pZH_AB4t+A6b_MMCas9-Nuclease											
callus No. (MF; DMC1A, DMC1B)		No.2 (56.2%, 46.6%)					No.3 (56.2%, 27.2%)				
Regenerated plants		1	2	3	4	5	1	2	3	4	5
On-target	DMC1A	M	M	M	M	M	N	N	N	B	N
Off-target	DMC1B	B	M	M	M	M	N	N	N	N	N

pZH_AB4t+A6b_MMCas9-Nickase																											
callus No. (MF; DMC1A, DMC1B)		No.1 (25%, N.D.)																			No.2 (21.8%, N.D.)						
Regenerated plants		1	2	3	4	5	6	7	8	9	10	11	12	13	14	15	16	17	18	19	1	2	3	4	5	6	7
On-target	DMC1A	M	N	M	M	M	N	N	N	N	N	M	M	B	N	M	M	B	M	N	N	M	N	B	N	N	M
Off-target	DMC1B	N	N	N	N	N	N	N	N	N	N	N	N	N	N	N	N	N	N	N	N	N	N	N	N	N	N

pZH_B1t+BA1b_MMCas9-Nuclease																																					
callus No.		No.1														No.2													No.3								
Regenerated plants		1	2	3	4	5	6	7	8	9	10	11	12	13	14	1	2	3	4	5	6	7	8	9	10	11	12	13	1	2	3	4	5	6	7	8	9
On-target	CDKB2	B	B	B	B	B	B	B	B	B	B	B	B	B	B	N	N	B	N	N	B	N	N	B	B	N	B	N	M	M	M	M	M	B	B	B	M
Off-target	CDKA2	B	B	B	B	B	B	B	B	B	B	B	B	B	B	N	N	B	N	N	B	N	N	B	B	N	B	N	M	B	M	M	M	B	B	B	M
	CDKB1	M	N	N	M	N	M	M	M	B	M	M	M	M	M	N	N	M	N	N	M	N	N	B	B	N	M	N	N	N	N	N	N	N	N	N	N
	CDKA1	N	N	N	N	N	N	N	N	M	N	N	N	N	N	N	N	M	N	N	N	N	N	M	M	N	N	N	N	N	N	N	N	N	N	N	N

pZH_B1t+BA1b_MMCas9-Nickase																								
callus No.		No.1										No.2								No.3				
Regenerated plants		1	2	3	4	5	6	7	8	9	10	1	2	3	4	5	6	7	8	1	2	3	4	5
On-target	CDKB2	M	M	M	M	M	M	M	M	M	M	M	M	M	M	M	M	M	M	N	N	M	N	N
Off-target	CDKA2	N	N	N	N	N	N	N	N	N	N	N	N	N	N	N	N	N	N	N	N	N	N	N
	CDKB1	N	N	N	N	N	N	N	N	N	N	N	N	N	N	N	N	N	N	N	N	N	N	N
	CDKA1	N	N	N	N	N	N	N	N	N	N	N	N	N	N	N	N	N	N	N	N	N	N	N

* Callus No. is the order of top three high mutated calli in total 24 lines.

B, bi-allelic mutation
M, mono-allelic mutation
N, non-mutation

○, deletion between paired gRNAs on mono-allelic
◎, deletion between paired gRNAs on bi-allelic
×, no- deletion between paired gRNAs

Table S3. List of primers used in this study.

Usage	Primer name	Sequence (5'→3')
gRNA-oligo	OsU6-A1t-F	GTTGTGCTCATTAGTTTGAATTCA
	A1t-R	AAACTGAATTCAAACTAATGAGCA
	OsU6-A2t-F	GTTGCCAGAGATGAACAGTAATGC
	A2t-R	AAACGCATTACTGTTCATCTCTGG
	OsU6-AB3b-F	GTTGTGGAGATGTGAAGAAGCTGC
	AB3b-R	AAACGCAGCTTCTTCACATCTCCA
	OsU6-AB4t-F	GTTGCCTTCTTTGTATGCATCATG
	AB4t-R	AAACCATGATGCATACAAAGAAGG
	OsU6-AB5b-F	GTTGCCTCATGATGCATACAAAGA
	AB5b-R	AAACTCTTTGTATGCATCATGAGG
	OsU6-A6b-F	GTTGTCTCCTGCTTGTTCCATTTC
	A6b-R	AAACGAAATGGAACAAGCAGGAGA
	OsU6-AB7b-F	GTTGTTCATCTTGTAGAGCCTGAC
	AB7b-R	AAACGTCAGGCTCTACAAGATGAA
	OsU6-A2t-1-F	GTTGCCGGAGATGAACAGTAATGC
	A2t-1-R	AAACGCATTACTGTTCATCTCCGG
	OsU6-A2t-2-F	GTTGCCAGAAATGAGCAGTAATGC
	A2t-2-R	AAACGCATTACTGCTCATTTCTGG
	OsU6-AB3b-1-F	GTTGTGAAGATGTGAAGAAGCTGC
	AB3b-1-R	AAACGCAGCTTCTTCACATCTTCA
	OsU6-AB3b-2-F	GTTGTGGAAATGTGGAGAAGCTGC
	AB3b-2-R	AAACGCAGCTTCTCCACATTTCCA
	OsU6-B1t-F	GTTGCCTTCTCCAGCTTCTCGTAC
	B1t-R	AAACGTACGAGAAGCTGGAGAAGG
	OsU6-B2t-F	GTTGGCCATCTCTCTCTCGCTGGT
	Bt2-R	AAACACCAGCGAGAGAGAGATGGC
	OsU6-BAb1-F	GTTGAGGTCGGGGAGGGGACGTAC
	BAb1-R	AAACGTACGTCCCCTCCCCGACCT
PCR	DMC1A-F	CATTAACCCGTCAAAATGATCGCTGGAACT
	DMC1A-R	AAGAATAAACCAAACAGCCCAGGGAAGTGC
	DMC1B-F	AGTCAATTTTACCCGCGTTTGGTGGAACTC
	DMC1B-R	ATGAAGCCCTGGCTCTGAGAAAAACAAACA
	CDKB2-F	AAACCCTAAATCCACGCGCATTCCACACCA
	CDKB2-R	TGGCAGAAAGCAACGCCCTTGCAGAGCTGGT
	CDKB1-F	ACGCTCCTCCCCCATTTCAAATC
	CDKB1-R	AGAAGCGGAGAAGACACGGGATAATCAGGCA
	CDKA2-F	ATGCCACAAGCCCAACCCAATTCATCCCCA
	CDKA2-R	TGCAGCCTGCACGGCACAATCCAAATTCCCA
	CDKA1-F	TCCTCTTCCTCCCACTTCTCCGCCTCCTCT
	CDKA1-R	TCAGGCAACCAATCAATCAGTCCGTGATGACT
